# Prevalence and Predictors of Anemia and Iron Deficiency in Children Aged 6 to 12 Years in Tunisia: A Nationwide Cross-Sectional Study

**DOI:** 10.3390/nu17213399

**Published:** 2025-10-29

**Authors:** Jalila El Ati, Radhouene Doggui, Besma Mourou, Myriam El Ati-Hellal

**Affiliations:** 1 SURVEN (Nutrition Surveillance and Epidemiology in Tunisia) Research Laboratory, INNTA (National Institute of Nutrition and Food Technology), 11 Rue Jebel Lakhdar, Bab Saadoun, Tunis 1007, Tunisia; jalila.elati@yahoo.fr (J.E.A.); doggui.radhouene@gmail.com (R.D.); 2 University Tunis El Manar, Tunis 1068, Tunisia; 3 Higher School of Health Sciences and Techniques of Tunis, Tunis 1007, Tunisia; besmamourou@yahoo.fr; 4 Laboratory Materials Molecules and Applications, Preparatory Institute for Scientific and Technical Studies, University of Carthage, B.P. 51 La Marsa, Tunis 2070, Tunisia

**Keywords:** anemia, iron deficiency, schoolchildren, risk factors, Tunisia

## Abstract

**Background**: Anemia and iron deficiency (ID) affect children and are regarded as a major public health problem in developing countries. This study aimed to evaluate the prevalences of anemia and ID in Tunisian children aged 6 to 12 years and to identify their associated risk factors. **Methods**: A nationwide cross-sectional survey was conducted across the seven major regions of Tunisia. A total of 2610 schoolchildren were selected using a two-stage random sampling method. Venous blood was collected to measure hemoglobin and ferritin for iron status determination. C-reactive protein (CRP) and Alpha-1-acid glycoprotein (AGP) were also measured to establish the inflammatory status of children. Multivariable logistic regression models were performed to identify factors associated with anemia and ID. **Results**: Anemia affected 5.7% of children, similarly between girls and boys. A quarter of children had ID and iron deficiency anemia (IDA) was diagnosed in 42% of the anaemic schoolchildren. In rural areas, girls were more affected by ID than boys whereas no significant difference was observed for anemia between both genders. Multivariate analysis revealed that insufficient consumption of iron-rich foods significantly increased the risk of ID (OR = 1.40; 95% CI [1.05–1.85]; *p* = 0.021). Children in public schools were 1.74 times more likely to be at risk of ID (95% CI [1.34–2.21]; *p* = 0.004) than those in private schools. **Conclusions**: To alleviate the burden of ID, national interventions should focus on iron supplementation, food diversification, nutritional education and regular longitudinal studies.

## 1. Introduction

Childhood and adolescence represent a crucial phase of rapid physical and cognitive growth, requiring adequate nutrition to support these processes. Numerous studies have demonstrated that micronutrient deficiencies during this period negatively impact growth, neuropsychological behavior, cognitive and motor development, intelligence quotient, attention, learning, memory, language abilities, and educational performance [1,2,3,4]. ID is one of the most serious micronutrient deficiencies worldwide as it is the leading cause of anemia, particularly in developing countries [5,6,7]. In Tunisia, ID is a major public health problem, mainly among women of childbearing age and children [8,9,10,11]. In 1975, anemia affected more than the third of the Tunisian population with a predominance in children under 2 years old, preschool children, women of childbearing age, pregnant women and breastfeeding women [8]. More than twenty years later, the prevalence of anemia has slightly decreased, but the same population groups remain the most affected [9]. A study on the causes of anemia in Tunisia, carried out in 2000 [10], showed that IDA accounted for more than two-thirds of anemia in the target population, children and women of childbearing age. The latest national health survey in 2016 highlighted a prevalence of anemia in the Tunisian population aged 15 years and over of 25.8% (17.0% in men and 34.3% in women). It was high among people aged 70 years and over (36.6%) [11].

Iron plays a crucial role in the development of children and adolescents. The most frequently observed symptoms of ID in children and adolescents include fatigue, delayed psychomotor and cognitive development, and decreased academic and athletic performance [12]. In children, especially girls at puberty, fatigue and weakness due to ID may contribute to absenteeism and school dropout. Currently, there are 100,000 school dropouts per year among Tunisian children in basic education (primary and secondary schools) [13]. These abnormalities are mostly evident in the presence of anemia [14]. In this age group, iron replacement has been shown to be effective, even in the absence of anemia, with an improvement in attention, concentration, and learning [13].

The main objective of this study was to estimate, at the national level, the prevalences of ID and anemia in children aged 6 to 12 years and to identify their associated demographic, dietary, socioeconomic, and environmental causes.

## 2. Materials and Methods

### 2.1. Subjects and Study Design

**Figure 1:**
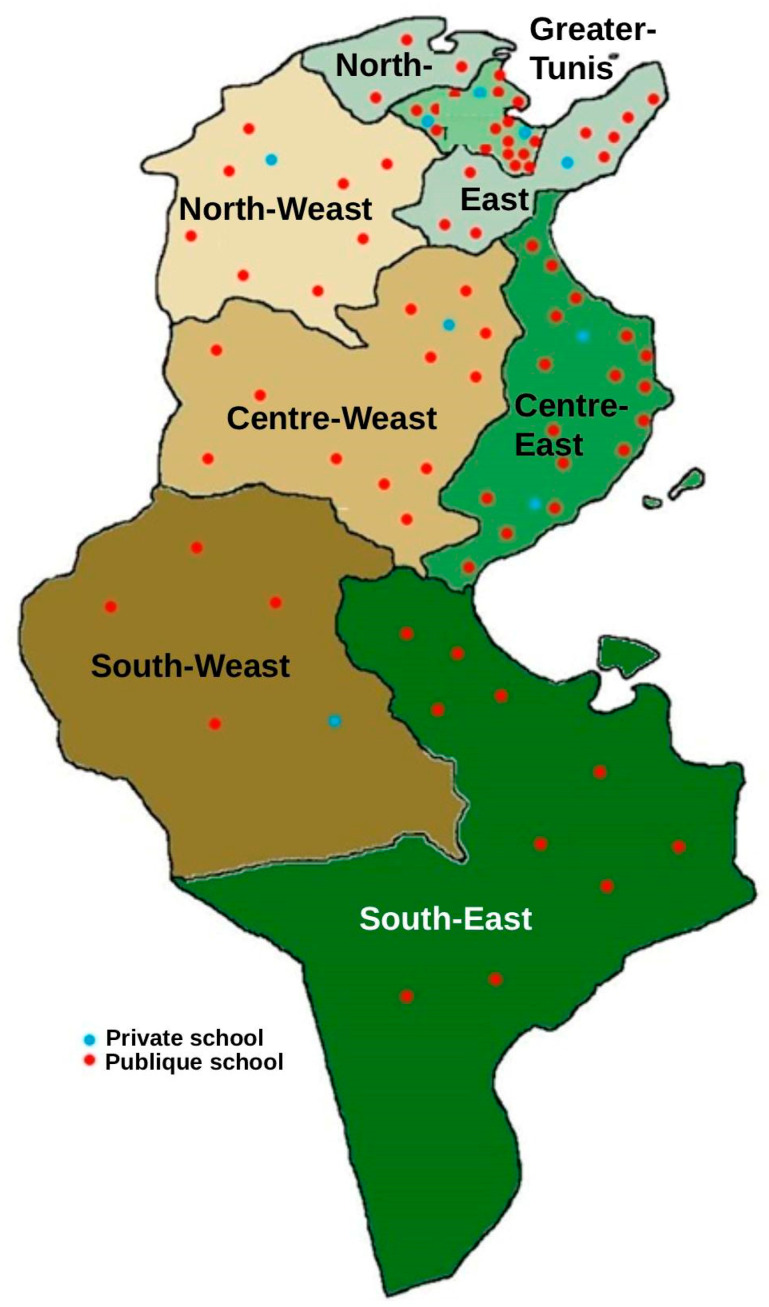
Distribution of schools by region and type of school (public/private).

From June to October 2023, a nationwide cross-sectional survey was conducted on a representative random sample of 87 primary schools, distributed proportionally across the seven major regions of Tunisia (Figure 1). From each school, a random sample of 30 students (15 boys and 15 girls) was selected, for a total of 2610 students aged 6 to 12 years.

The survey plan and sampling were carried out by the Ministry of Education, the General Directorate of Studies and Planning, in agreement with specialists from the National Institute of Statistics. The sample was drawn using a two-stage random sampling method. In the first stage, a random selection of 87 primary schools was conducted proportionally to their size, stratified first by the seven major regions, then by residential area, and finally by public or private school type. In the second stage, a systematic selection of 30 students, stratified by age and gender, was conducted from the list of students enrolled in each school for the 2022–2023 school year. The inclusion criteria were as follows: children aged 6 to 12, enrolled in school and who consented to participate in the study and whose parents or legal guardians also provided their consent. No specific exclusion criteria were defined, other than not meeting the inclusion criteria.

### 2.2. Data Collection

#### 2.2.1. Anthropometric Characteristics

Weight and height were measured on the day of the survey. Height was measured to the nearest 0.1 cm using a height rod fixed to a vertical board and equipped with a measuring tape graduated to the nearest mm and a movable horizontal cursor (Person-chek^®^, Stuttgart, Germany). Weight was determined by an electronic scale (Seca 770, Hamburg, Germany) to the nearest 0.1 kg. Quality control of anthropometric data was carried out in accordance with WHO recommendations by identifying improbable measurements [15]. Body mass index (BMI) was used to assess the nutritional status of children aged 6 to 12 years. It is expressed as a number of standard deviations or z-scores below or above the median of the reference populations by age and gender, defined by the WHO [16]. The height-age index was calculated with reference to the WHO International Growth Standards [17] and expressed as a number of z-scores, relative to the median of the population by age and gender.

#### 2.2.2. Iron-Rich Food Intake

To estimate the consumption of iron-rich foods, we used a semi-quantitative food frequency questionnaire (FFQ) with a recall period of one month preceding the survey date. Although the FFQ did not explicitly account for seasonal variations in food availability, the recall period was chosen to capture usual intake over a sufficiently long period, thus minimizing short-term fluctuations. The list of foods included in the questionnaire was developed following the method proposed by Morabia (1994) [18], which identifies foods contributing to at least 90% of total iron intake within a comparable population group. This list was derived from data from the most recent dietary survey conducted in 2019 among schoolchildren aged 6 to 9 years living in Greater Tunis [18,19,20]. To estimate quantities consumed, the distribution of portion sizes recorded in the 2019 survey was used to establish three standard portion categories for each food item: (i) reference portion: median portion size; (ii) small portion: first quartile (Q1); large portion: third quartile (Q3).

During data collection, the mothers were asked to indicate whether their child’s consumption was smaller, equal to, or larger than the reference portion. Accordingly, the “small”, “reference”, or “large” portion was selected. Standard household measures were used to estimate portion weights. Consumption frequency was assessed over the month preceding the survey using a nine-level scale ranging from “never or less than once a month” to “four or more times per day”.

#### 2.2.3. Socioeconomic Characteristics

The three main indicators of socioeconomic variables are income, education and occupation of the parents. The household’s economic level was assessed from a questionnaire sent to the parents or guardians according to a methodology used several times in the Tunisian context [21,22,23]. Information on parents’ educational level was also collected through telephone interviews to assess socioeconomic status. Their professional activity was classified into three categories: senior or middle managers, employees or workers, without activity. The activity of the head of household was classified into two categories (with professional activity, without professional activity).

#### 2.2.4. Iron Status Assessment

An aliquot of venous blood samples was collected by trained biologists or nurses for on-site hemoglobin measurement using a portable hemoglobinometer (HemoCue^®^ Hb 201+, HemoCue AB, Ängelholm, Sweden), which is calibrated according to the cyan methemoglobin (HiCN) standard method [24]. Hemoglobin was analyzed for the diagnosis of anemia, a pathological condition in which the number of red blood cells is insufficient to meet the physiological needs of the body. It is defined as a circulating level of hemoglobin below the limits set by the WHO [25,26]. The thresholds for anemia and its different types in children aged 6 to 12 years are presented in Table 1.

To obtain the serum, another venous blood aliquot was centrifuged at 3500 rpm for 10 min after coagulation. Tubes containing serum aliquots (between 50 and 100 μL each) were shipped to the laboratory of the Hohenheim University, at the Institute of Biological Chemistry and Nutrition in Stuttgart, Germany, where a single ELISA test was developed to measure plasma ferritin for iron status determination [27]. CRP and AGP were also measured to establish the inflammatory status of children and to correct the markers of iron status. Indeed, inflammation and nutrition are closely linked, resulting in the alteration of many nutritional biomarkers [28]. Specifically, CRP levels begin to decrease between 24 and 48 h after the onset of inflammation, while AGP levels remain elevated for 5 to 6 days and ferritin levels for up to 10 days [28]. “Inflammation” is defined if the concentration of CRP exceeds 5 mg/L and/or that of AGP exceeds 1 g/L. “Iron deficiency” is defined if serum ferritin concentration is under 15 μg/L for children aged 6–12 years [29]. In the absence of inflammation, ferritin concentration is positively correlated with the magnitude of total iron stores, and hence the WHO recommends its use in the assessment of iron status. However, since ferritin is an acute-phase protein, its concentration increases during the inflammatory process and therefore, it no longer reflects iron stores. In this case, adjustments are necessary, but there is no consensus on a method for correcting inflammation.

The method adopted in our study, was developed by Thurnham et al. (2010) [30]. It involves stratifying individuals into categories based on elevated CRP and/or AGP: apparently healthy reference group (CRP ≤ 5 mg/L and AGP ≤ 1 g/L), incubating group (CRP > 5 mg/L and AGP > 1 g/L), early convalescence group (CRP > 5 mg/L and AGP ≤ 1 g/L), and late convalescence group (CRP ≤ 5 mg/L and AGP > 1 g/L). Individual values were then adjusted with multiplication by Thurnham correction factors (0.77, 0.53, and 0.75, respectively), which were obtained from the ratio of the geometric mean of the apparently healthy group to the geometric mean of each inflammatory group [30].

### 2.3. Statistical Analysis

Data analysis was performed using Stata 14 software (StataCorp, College Station, TX, USA, 2015), taking into account the complex sampling design (including stratification, sampling weights, and post-stratification on sex, age, place of residence, and school type) and using svy Stata commands specific to survey data analysis [31]. Results were presented as means and standard error of the mean for continuous variables, and as percentages for categorical variables and the 95% confidence interval. The association between two categorical variables was assessed by the Chi-square test. Logistic regression was used to examine the association between iron deficiency and the explanatory variables. The first-type risk threshold was set at 0.05 for all analyses.

### 2.4. Ethics

The study protocol has obtained ethical approval from the Human Research Ethics Committee of INNTA (Visa No. 22/2022) of 20 December 2022, and from the National Council of Statistics of Tunisia, (Visa No. 03/2023). The National Authority for the Protection of Personal Data (INPDP) has given 3 favorable opinions for the transfer of data abroad (22/02-7064), health data (22/02-7063) and personal data (22/01-1539) and the family judge has given a favorable opinion for the study (No. 15152 of 14 December 2022).

## 3. Results

In practice, 2670 children were surveyed, corresponding to an overall response rate of 102.3% (104.4% for girls and 100.0% for boys). This rate exceeding 100% resulted from the inclusion of approximately 60 additional pupils who met the eligibility criteria and whose parents expressed a strong desire for their participation. Their inclusion did not alter the sample’s representativeness, as the selection remained proportionally distributed by region, residence area (urban/rural), school type (public/private), age, and sex. Therefore, the risk of selection bias is negligible, particularly since participation among children from both public and private schools was complete.

Table 2 represents the demographic and socioeconomic data. The national survey was conducted among 2670 schoolchildren (1363 girls and 1307 boys), aged 6 to 12 years (mean age 9.39 ± 0.05 years for boys and 9.50 ± 0.05 years for girls; *p* = 0.16) selected for the study. The response rate was 100% for both genders. Among the schoolchildren, 70.2% lived in urban areas and 29.8% in rural areas, which shows good representativeness according to the living area of the studied sample.

The educational level of parents or guardians was comparable for both genders. Just over a third of mothers and a quarter of fathers had completed secondary education and pursued higher education, while only 7.4% of mothers and 3.3% of fathers had never attended school. Of all mothers, only 26.4% were employed. Three-quarters of mothers were housewives, while 6.8% of fathers were not employed. A quarter of fathers and 13.7% of mothers were middle or senior managers.

Table 3 presents the mean values of biological markers, namely circulating CRP, AGP, hemoglobin and ferritin. The mean values of the inflammatory markers, CRP and AGP, showed no significant differences between girls and boys, whether at the national level, by residential area, or by region. The mean values of these markers were below the thresholds for inflammation, namely 5 mg/L for CRP and 1 g/L for AGP.

Mean hemoglobin levels were similar between both genders, whether at the national, urban–rural, or regional levels. The average values were above the thresholds for anemia for this population group, namely 115 g/L for children aged 6 to 11 years and 120 g/L for children aged 12 years (Table 1). Serum ferritin corrected for inflammatory markers was similar between boys and girls. Mean values were above the thresholds for iron deficiency (15 µg/L), whether at the national, regional, or local level.

Table 4 displays the prevalence of ID and anemia and the distribution of the anemia’s degree of severity by gender, living area and region. A quarter of the children had ID and there was no significant difference between girls and boys. According to the living area, the prevalences were equally distributed between urban and rural areas. However, in rural areas, girls were more affected than boys (18.9% with 95% CI [15.1–23.3] versus 26.5% with 95% CI [21.7–31.8]; *p* = 0.014). The prevalences were statistically equal between regions (*p* = 0.37). They varied between 18.6% (with 95% CI [12.7–26.4) in the North-West and 27.7% (with 95% CI [23.1–32.8]) in the Centre-East and 27.7% (with 95% CI [23.2–32.8]) in Greater Tunis.

Nationally, anemia affected 5.7% (95% CI [4.6–7.1]) of children aged 6–12 years, similarly between girls (6.5% with 95% CI [5.0–8.5]) and boys (4.9% with 95% CI [3.6–6.7]). At the living area level, there was no significant difference in the prevalence of anemia. Moreover, anemia was distributed equally between girls and boys in each living area. At the regional level, no significant difference was observed either between regions or between both genders within each region.

Severe anemia was relatively rare nationally. It affected only 0.1% of children (95% CI [0.1–0.4]) and was equally distributed between girls (3.0% with 95% CI [2.1–4.2]) and boys (2.4% with 95% CI [1.5–3.8]). At the local and regional levels, severe anemia was rare to nonexistent. Moderate anemia affected 2.7% (95% CI [2.2–5.1]) of the schoolchildren and similarly in girls (3.0% with 95% CI [2.1–4.2]) and boys (2.4% with 95% CI [1.5–3.8]; *p* = 0.45). No difference was observed between urban and rural areas and between the seven Tunisian regions. At the level of each living area and each region, the prevalence of anemia was equally distributed between the two genders. Mild anemia affected 2.9% (95% CI [2.2–3.8]) of children aged 6–12 years at the national level. No gender differences were observed for mild anemia at the national level, in each living area and in each region. The prevalence of mild anemia showed no significant difference between urban and rural settings, and between the seven major administrative regions.

In univariate analysis, both ID and anemia were significantly associated with low consumption of iron-rich foods. Furthermore, children attending public schools or whose mothers held middle or senior management positions were at increased risk of ID. Thinness appeared to protect children against ID (Table 5).

Multivariate analysis confirmed that insufficient consumption of iron-rich foods significantly increased the risk of ID (OR = 1.40; 95% CI [1.05–1.85]; *p* = 0.021). However, it didn’t affect anemia (OR = 0.99; 95% CI [0.43–2.25]; *p* = 0.97). Children in public schools were 1.74 times more likely to be at risk of ID (95% CI [1.34–2.21]; *p* = 0.004) than those in private schools. The other studied variables were not significantly associated with ID or anemia (Table 5).

## 4. Discussion

In our study, the overall prevalence of anemia in schoolchildren aged 6 to 12 years was 5.7%. Anemia was distributed similarly between girls and boys at the national level, according to living areas and regions. Neither inter-area nor inter-regional differences were observed. The prevalence of anemia in Tunisian school aged children is considerably lower to the estimated global prevalence for the same population group, which sits around 25.4% [32]. It is similar to that reported by the national nutrition survey in 1996–97 (5.7% vs. 6.4%) [9]. Despite the fact that the prevalences are low, no significant improvement has been recorded in recent decades. In general, preschool children and women of childbearing age are the vulnerable groups for this pathology due to their important physiological needs related to growth and gestation respectively [33,34]. Comparable prevalences were also reported among schoolchildren in Colombia (5.7%), China (5.7%), Costa Rica (6%), Panama (6.3%), Vietnam (6.8%), Ecuador (4.8%), the United Kingdom (4.8%) and the United States (4.8%) [35,36,37]. Higher prevalences were registered among school aged children in Bangladesh (42.1%) [38], Ethiopia (23%) [39], Ghana (23.5%) [40], Egypt (35.3%) [41], India (54.2%) [42], South Africa (13.8%), Tanzania (46%) [43], Thailand (48.8%) [44] and Morocco (12.2%) [45]. According to Wrottesley et al. (2023) who evaluated the nutritional status of school-age children and adolescents in low- and middle-income countries across seven global regions, the most affected region by anemia is West and Central Africa [46]. However, the highest anemia prevalence among the same population group was registered in Indonesia (98.5%) [46]. Werner et al. (2025) examined the burden of anemia in children aged 5 to 19 years from 17 surveys in 16 countries and found a median overall anemia prevalence of 16% [35]. In most surveys, no gender differences were observed and anemia was more prevalent among children aged 15 to 19 (22%), followed by those aged 5 to 9 years (9%) and finally those aged 10 to 14 years (7%).

ID was observed in a quarter of children. It was equally distributed between girls and boys at the national level. No significant differences were observed between areas and regions. Gender disparity was observed only in rural areas where girls were more affected than boys, probably because girls lose blood during menstruation and have poorer socio-economic conditions in rural areas. The prevalence of ID among schoolchildren in our study is higher than that documented in studies from Bangladesh (4.6%), Colombia (8.5%), Ecuador (2.3%), Malawi (4.2%) [35], Lebanon (14.2%) [47], Malaysia (5.2%) [48] and China (6.2%) [49], similar to that reported in Spain (25%) [50] and lower than that registered in Egypt (38.2%) [41], Ghana (71.3%) [46] and South Africa (27.7%) [51]. Our results revealed that IDA was diagnosed in 42% of the anaemic schoolchildren, with a global prevalence of 2.4%. These results suggest that ID is an important factor of anemia in Tunisian schoolchildren. Similar to our study, Andriastuti et al. (2020) found ID as the cause of anemia in 44.4% anaemic children and adolescents from Indonesia [6]. Werner et al. (2025) reported that ID was the most consistent determinant of anemia among the same population group [35]. According to the WHO, almost 50% of anemia could be caused by ID worldwide [52]. Other possible anemia predictors could be chronic kidney disease, inflammation, haemoglobinopathies, malaria, infectious diseases, micronutrient deficiencies (vitamin A, folates, vitamin B12, zinc), BMI, and gynaecological disorders for women in childbearing age [35,53].

From multivariable logistic regression analysis, a significant association is obtained between ID and the consumption of iron-rich foods. These findings highlight the need for a program to reduce iron deficiency in children. This program should include interventions to promote the consumption of iron-rich animal foods, such as meat, poultry, and fish (sources of heme iron), plant-based foods such as cereals, pulses, legumes, fruits, and vegetables (sources of non-heme iron), and foods rich in vitamin C to stimulate the absorption of non-heme iron [54,55].

Children enrolled in public schools were at higher risk of iron deficiency compared to those enrolled in private schools. In Tunisia, school feeding programs play a key role in improving nutrition and access to education for children from disadvantaged households. These programs aim to reduce school dropout rates, often linked to the distance from schools, and to improve children’s concentration and learning abilities by combating hunger. Managed by the Ministry of Education, particularly through the Office of School Works, these programs involve various actors, such as local farmers, professional agricultural organizations, and production cooperatives. They cover the entire process, from menu planning to meal distribution. However, these initiatives face logistical challenges, including fragmented supply chains and the mismatch between local production and canteen needs. These challenges are exacerbated by limited infrastructure, such as a lack of adequate storage and poorly equipped kitchens.

## 5. Strengths and Limitations of the Study

One of the strengths of this study is that the sample was representative of the whole primary schoolchildren population in Tunisia, thus providing a nationwide database. Furthermore, the assessment of ID was based on ferritin concentration adjusted for inflammation, which allows an accurate estimate of the body’s iron reserves. However, the study has some limitations. Firstly, the cross-sectional design means the temporality between anemia, ID and their model predictors cannot be established. Secondly, we used a retrospective collection method which may have introduced some recall bias when answering the semi-quantitative questionnaire. Finally, we didn’t evaluate the effects of other potential predictors of anemia such as parasitic infections and diseases, and haemoglobinopathies.

## 6. Conclusions

This national study revealed that one in four Tunisian schoolchildren aged 6 to 12 years suffered from iron deficiency, with notable disparities among girls living in rural areas and children attending public schools, who were the most affected groups. Anemia affected 5.7% of children, with no significant difference between sexes or regions, and iron deficiency anemia (IDA) accounted for 42% of anemia cases.

To effectively reduce the burden of iron deficiency in Tunisia, interventions should be specifically directed toward the identified high-risk groups. In particular, school-based iron supplementation program should be prioritized in public schools, which host the majority of children from lower socioeconomic backgrounds. In rural areas, actions should focus on enhancing food diversification by improving access to and utilization of locally produced, iron-rich foods such as legumes, pulses, fish, and fortified cereals.

Furthermore, nutrition education and awareness campaigns targeting rural families and adolescent girls are needed to promote the consumption of iron-rich foods and foods that enhance iron absorption, such as those rich in vitamin C. Strengthening school feeding programs and integrating local agricultural value chains could also contribute to sustainable improvements in children’s nutritional status.

Finally, establishing regular monitoring and longitudinal studies will be essential to evaluate the long-term effectiveness of these targeted interventions and to adapt strategies to evolving nutritional and socioeconomic contexts.

## Figures and Tables

**Table 1:** Different types of anemia and their thresholds in children aged 6 to 12 years.

Age of Children	Hemoglobin Concentration (g/L)				
	No Anemia	Anemia	Mild Anemia	Moderate Anemia	Severe Anemia
6 to 11 years	115 or more	<115	110–114	80–109	<80
12 years	120 or more	<120	110–119	80–109	<80

**Table 2:** Sociodemographic characteristics of Tunisian children aged 6 to 12 years classified by gender.

	Girls	Boys	Girls vs. Boys
	% ^1^ [95% CI ^2^]	% ^1^ [95% CI ^2^]	*p* ^3^
Age (years)			
<10	58.2 [55.6–60.7]	60.3 [57.3–63.3]	0.23
≥10	41.8 [39.3–44.4]	39.7 [36.8–42.8]	
Living area			
Urban	69.6 [58.5–78.8]	70.9 [60.1–79.8]	0.31
Rural	30.4 [21.2–41.5]	28.1 [20.3–69.9]	
Region			
Greater-Tunis	20.9 [19.0–22.8]	22.0 [20.3–23.8]	0.42
North-East	13.9 [12.8–15.2]	14.1 [13.1–15.1]	
North-West	9.8 [7.9–12.0]	8.7 [7.0–10.7]	
Centre-East	16.0 [14.5–17.6]	16.5 [15.1–17.9]	
Centre-West	15.2 [13.0–17.8]	14.8 [16.5–16.3]	
South-East	17.8 [14.9–21.1]	18.5 [15.7–21.6]	
South-West	6.4 [5.1–8.2]	5.4 [4.6–6.5]	
Father’s education			
Secondary or more schooling	30.2 [27.9 [32.6]	26.3 [23.7–29.0]	0.062
Uncompleted secondary schooling	67.2 [64.5–69.6]	69.9 [67.3–72.3]	
No formal schooling or Primary schooling	2.6 [1.9–3.7]	3.9 [2.9–5.2]	
Mother’s education			
Secondary or more schooling	37.9 [35.0–41.0]	36.0 [32.7–39.4]	0.65
Uncompleted secondary schooling	57.6 [51.5–57.6]	56.8 [53.3–60.2]	
No formal schooling or Primary schooling	7.5 [6.2–9.1]	7.2 [5.7–9.2]	
Father’s occupation			
Middle/upper executive	23.4 [20.0–27.3]	24.7 [21.0–28.8]	0.58
Worker/employee	69.4 [65.4–73.1]	68.9 [65.1–72.5]	
Not working	7.2 [5.4–9.5]	6.4 [4.9–8.2]	
Mother’s occupation			
Middle/upper executive	15.2 [12.2–18.8]	12.1 [9.7–15.1]	0.049
Worker/employee	12.2 [9.9–15.0]	13.3 [10.8–16.3]	
Not working	72.6 [68.2–76.6]	74.6 [70.9–77.9]	

^1^ Weighted proportions accounting for sampling design. ^2^ 95% confidence interval taking into account sampling design. ^3^ *p*-value of the comparison of Girls versus Boys (chi2 test).

**Table 3:** Biological characteristics of Tunisian children aged 6 to 12 years classified by gender, area and region.

	CRP ^1^ (mg/L) Mean (SD) ^3^	AGP ^2^ (g/L) Mean (SD) ^3^	Hemoglobin (g/L) Mean (SD) ^3^	Corrected Ferritin (µg/L) Mean (SD) ^3^
	(*n* = 2670)	(*n* = 2670)	(*n* = 2670)	(*n* = 2670)
National				
National	*p* ^4^ = 0.18	*p* ^4^ = 0.054	*p* ^4^ = 0.11	*p* ^4^ = 0.22
Girls	1.33 (0.11)	0.80 (0.01)	13.18 (0.05)	26.41 (0.61)
Boys	1.13 (0.10)	0.82 (0.01)	13.25 (0.05)	27.50 (0.72)
Total	1.23 (0.08)	0.81 (0.01)	13.22 (0.05)	26.92 (0.52)
Living area				
Urban	*p* ^4^ = 0.13	*p* ^4^ = 0.39	*p* ^4^ = 0.15	*p* ^4^ = 0.21
Girls	1.18 (0.13)	0.82 (0.01)	13.10 (0.05)	25.53 (0.62)
Boys	1.52 (0.16)	0.83 (0.01)	13.19 (0.05)	26.63 (0.93)
Total	1.36 (0.11)	0.82 (0.01)	13.14 (0.04)	26.08 (0.64)
Rural	*p* ^4^ = 0.79	*p* ^4^ = 0.059	*p* ^4^ = 0.36	*p* ^4^ = 0.70
Girls	0.90 (0.12)	0.76 (0.01)	13.36 (0.14)	28.41 (1.44)
Boys	0.97 (0.14)	0.80 (0.01)	13.41 (0.11)	29.61 (0.76)
Total	0.94 (0.10)	0.77 (0.01)	13.38 (0.12)	28.99 (0.92)
Region				
Greater-Tunis	*p* ^4^ = 0.053	*p* ^4^ = 0.34	*p* ^4^ = 0.19	*p* ^4^ = 0.066
Girls	1.39 (0.30)	0.86 (0.02)	13.06 (0.11)	24.15 (1.09)
Boys	1.22 (0.21)	0.87 (0.03)	13.20 (0.10)	28.00 (2.28)
Total	1.80 (0.25)	0.87 (0.02)	13.13 (0.10)	26.08 (1.43)
North-East	*p* ^4^ = 0.072	*p* ^4^ = 0.93	*p* ^4^ = 0.38	*p* ^4^ = 0.30
Girls	1.07 (0.20)	0.84 (0.02)	13.42 (0.17)	26.52 (1.30)
Boys	1.76 (0.33)	0.82 (0.02)	13.31 (0.16)	28.29 (1.36)
Total	1.42 (0.16)	0.83 (0.02)	13.36 (0.15)	27.42 (1.11)
Noth-West	*p* ^4^ = 0.11	*p* ^4^ = 0.12	*p* ^4^ = 0.17	*p* ^4^ = 0.47
Girls	0.95 (0.20)	0.78 (0.02)	13.25 (0.20)	29.20 (2.06)
Boys	0.65 (0.11)	0.79 (0.03)	13.48 (0.19)	28.51 (1.68)
Total	0.81 (0.13)	0.78 (0.02)	13.36 (0.17)	28.88 (1.52)
Centre-East	*p* ^4^ = 0.52	*p* ^4^ = 0.18	*p* ^4^ = 0.27	*p* ^4^ = 0.24
Girls	1.25 (0.20)	0.79 (0.01)	13.26 (0.09)	24.95 (0.95)
Boys	1.49 (0.32)	0.82 (0.02)	13.35 (0.06)	26.72 (1.35)
Total	1.37 (0.19)	0.81 (0.01)	13.30 (0.07)	25.83 (1.01)
Centre-West	*p* ^4^ = 0.52	*p* ^4^ = 0.001	*p* ^4^ = 0.83	*p* ^4^ = 0.99
Girls	0.86 (0.23)	0.75 (0.02)	13.35 (0.19)	29.71 (1.81)
Boys	1.1 (0.16)	0.84 (0.02)	13.34 (0.16)	29.73 (1.59)
Total	0.96 (0.14)	0.79 (0.02)	13.34 (0.16)	29.72 (1.37)
South-East	*p* ^4^ = 0.45	*p* ^4^ = 0.17	*p* ^4^ = 0.34	*p* ^4^ = 0.26
Girls	0.66 (0.09)	0.75 (0.02)	12.87 (0.10)	23.12 (1.68)
Boys	0.89 (0.29)	0.78 (0.02)	12.98 (0.10)	25.25 (1.45)
Total	0.78 (0.15)	0.77 (0.02)	12.93 (0.10)	24.18 (0.92)
South-West	*p* ^4^ = 0.35	*p* ^4^ = 0.92	*p* ^4^ = 0.15	*p* ^4^ = 0.72
Girls	0.72 (0.15)	0.74 (0.03)	13.15 (0.26)	30.32 (2.39)
Boys	1.50 (0.62)	0.76 (0.02)	13.40 (0.14)	30.33 (3.59)
Total	1.07 (0.24)	0.75 (0.02)	13.26 (0.19)	30.32 (2.87)

^1^ C-reactive protein. ^2^ Alpha1-acid glycoprotein. ^3^ Weighted mean (standard error of the mean). ^4^ *p* value Girls versus Boys.

**Table 4:** Prevalence of ID, and anemia and distribution of anemia’s degree of severity according to gender, area and region among Tunisian schoolchildren aged 6 to 12 years.

		ID ^1^	Girls vs. Boys	Anemia	Mild Anemia	Moderate Anemia	Severe Anemia	Girls vs. Boys
		% ^2^ [IC 95%] ^3^	*p* ^3^	% ^2^ [IC 95%] ^3^	% ^2^ [IC 95%] ^3^	% ^2^ [IC 95%] ^3^	% ^2^ [IC 95%] ^3^	*p* ^4^
National								
Girls		26.4 [23.7–29.4]	0.14	6.5 [5.0–8.5]	3.4 [2.2–5.1]	3.0 [2.1–4.2]	0.2 [0.1–0.6]	0.45
Boys		23.7 [21.0–26.7]		4.9 [3.6–6.7]	2.5 [1.7–3.6]	2.4 [1.5–3.8]	0.1 [0.0–0.6]	
Total		25.1 [23.0–27.4]		5.7 [4.6–7.1]	2.9 [2.2–3.8]	2.7 [2.2–5.1]	0.1 [0.1–0.4]	
Living area								
Urban	Girls	26.4 [23.2–29.9]	0.71	6.1 [4.5–8.3]	2.5 [1.5–4.1]	2.6 [1.5–4.4]	0.1 [0.0–0.7]	0.76
	Boys	25.7 [22.4–29.4]		5.4 [3.9–7.5]	2.7 [1.8–4.0]	3.5 [2.4–5.0]	0.1 [0.0–0.8]	
	Total	26.1 [23.4–29.0]		5.8 [4.5–7.3]	2.6 [1.9–3.5]	3.1 [2.1–4.4]	0.1 [0.0–0.4]	
Rural	Girls	26.5 [21.7–31.8]	0.014	7.5 [4.6–12.0]	5.3 [2.7–9.9]	1.8 [0.6–5.6]	0.4 [0.1–1.6]	0.19
	Boys	18.9 [15.1–23.3]		3.8 [1.6–8.7]	2.0 [0.8–4.9]	1.8 [0.8–3.8]	0.0	
	Total	22.8 [19.6–26.4]		5.7 [3.5–9.1]	3.7 [2.2–6.2]	1.8 [0.9–3.4]	0.2 [0.0–0.8]	
Region								
Greater-Tunis	Girls	29.6 [23.2–37.0]	0.36	8.7 [4.7–15.6]	5.8 [2.3–14.1]	3.0 [1.4–6.1]	0.0	0.32
	Boys	25.9 [25.0–32.0]		6.6 [4.0–10.5]	2.6 [1.4–4.9]	3.6 [1.7–7.1]	0.4 [0.0–3.0]	
	Total	27.7 [23.2–32.8]		7.6 [4.9–11.7]	4.2 [2.2–7.9]	3.3 [2.0–5.3]	0.2 [0.0–1.5]	
North-East	Girls	25.9 [19.1–34.2]	0.56	4.3 [1.8–9.9]	1.6 [0.5–5.2]	2.7 [0.9–7.3]	0.0	0.92
	Boys	23.3 [17.6–30.2]		4.0 [1.1–13.2]	1.7 [0.6–5.2]	2.3 [0.4–11.5]	0.0	
	Total	24.6 [19.8–30.2]		4.1 [1.9–8.8]	1.7 [0.7–3.9]	2.5 [0.8–7.2]	0.0	
North-West	Girls	18.7 [12.8–26.5]	0.95	4.5 [1.6–12.2]	3.4 [0.7–14.7]	1.0 [0.1–6.8]	0.0	0.28
	Boys	18.4 [10.5–30.2]		4.5 [1.0–18.3]	1.5 [0.3–7.0]	3.0 [1.2–7.4]	0.0	
	Total	18.6 [12.7–26.4]		4.5 [1.7–11.7]	2.4 [0.7–8.2]	2.1 [0.8–5.3]	0.0	
Centre-East	Girls	28.9 [22.6–36.1]	0.49	3.5 [2.0–6.1]	1.6 [0.5–5.0]	1.6 [0.7–3.5]	0.3 [0.0–2.1]	0.78
	Boys	26.5 [21.5–32.2]		3.7 [1.9–7.1]	2.3 [0.9–5.8]	1.4 [0.4–4.5]	0.0	
	Total	27.7 [23.1–32.8]		3.6 [2.5–5.0]	2.0 [1.0–3.7]	1.5 [0.7–2.9]	0.1 [0.0–1.1]	
Centre-West	Girls	23.8 [20.3–27.7]	0.97	6.1 [3.5–10.5]	2.8 [1.0–7.5]	2.5 [1.0–5.4]	0.9 [0.2–4.1]	0.37
	Boys	23.7 [17.3–31.5]		3.4 [1.4–8.3]	2.5 [0.9–6.8]	0.9 [0.2–4.0]	0.0	
	Total	23.7 [20.0–28.0]		4.8 [3.0–7.6]	2.7 [1.4–5.0]	1.6 [0.7–3.6]	0.5 [0.1–2.1]	
South-East	Girls	30.1 [20.0–42.4]	0.32	10.2 [5.3–18.7]	4.6 [2.0–10.1]	5.6 [2.4–12.7]	0.0	0.63
	Boys	23.6 [16.2–33.0]		7.1 [2.9–16.0]	2.6 [0.7–8.5]	4.5 [1.5–12.7]	0.0	
	Total	26.8 [20.2–34.7]		8.6 [4.9–14.8]	3.6 [2.1–6.2]	5.1 [2.3–10.8]	0.0	
South-West	Girls	19.2 [7.8–40.2]	0.84	5.5 [1.3–20.1]	4.1 [0.6–24.4]	1.4 [0.0–32.5]	0.0	0.54
	Boys	16.8 [1.0–79.8]		2.2 [0.2–23.8]	2.2 [0.2–23.8]	0.0	0.0	
	Total	18.1 [3.4–55.5]		4.0 [1.0–23.8]	3.3 [0.8–12.8]	0.8 [0.0–20.2]	0.0	

^1^ Iron Deficiency. ^2^ Prevalences weighted by extrapolation factors. ^3^ 95% confidence interval. ^4^ *p*-value of the comparison Girls versus Boys (chi2 test).

**Table 5:** Multivariable logistic regression analysis for the association of different variables with ID and anemia among Tunisian schoolchildren aged 6 to 12 years.

	Anemia				ID ^1^			
	Non-Adjusted		Adjusted		Non-Adjusted		Adjusted	
	OR ^2^ [IC 95% ^3^]	*p* ^4^	OR ^2^ [IC 95% ^3^]	*p* ^4^	OR ^2^ [IC 95% ^3^]	*p* ^4^	OR ^2^ [IC 95% ^3^]	*p* ^4^
Age (years)								
6–9	1.37 [0.97–1.92]	0.70	1.17 [0.78–1.77]	0.44	1.19 [0.96–1.44]	0.085	1.02 [0.79–1.31]	0.90
10–12	1	---	1	---	1	---	1	---
Gender								
Boy	1	---	1	---	1	---	1	---
Girl	1.35 [0.90–2.05]	0.14	1.40 [0.93–2.10]	0.11	1.16 [0.95–1.40]	0.14	1.16 [0.95–1.41]	0.15
Living area								
Urban	1.01 [0.56–1.80]	0.97	0.97 [0.49–1.92]	0.93	1.19 [0.93–1.52]		1.04 [0.81–1.33]	0.75
Rural	1	---	1	---	1	--	1	---
Region								
Greater Tunis	1.98 [0.71–5.50]	0.19	2.00 [0.66–6.07]	0.22	1.73 [0.57–5.21]	0.32	1.57 [0.51–4.88]	0.43
North East	1.03 [0.32–3.33]	0.96	1.00 [0.29–3.51]	0.99	1.47 [0.49–4.47]	0.49	1.54 [0.51–4.68]	0.44
North West	1.12 [0.31–4.05]	0.85	0.96 [0.26–3.61]	0.95	1.03 [0.33–3.24]	0.96	1.05 [0.34–3.27]	0.93
Centre East	0.89 [0.33–2.36]	0.81	0.81 [0.27–2.38]	0.69	1.73 [0.57–5.22]	0.33	1.68 [0.55–5.14]	0.36
Centre West	1.20 [0.43–3.35]	0.72	0.97 [0.30–3.15]	0.97	1.40 [0.47–4.21]	0.54	1.43 [0.49–4.21]	0.51
South East	2.26 [0.78–6.59]	0.13	2.25 [0.68–7.43]	0.18	1.65 [0.53–5.12]	0.38	1.75 [0.56–5.49]	0.33
South West	1	---	1	---	1	---	1	---
Iron-rich food intake								
Low	1.51 [0.96–2.37]	0.072	0.99 [0.43–2.25]	0.97	1.45 [1.16–1.81]	0.001	1.40 [1.05–1.85]	0.021
Middle	1.00 [0.62–1.62]	0.99	0.74 [0.39–1.40]	0.36	1.15 [0.89–1.48]	0.27	1.12 [0.84–1.49]	0.441
High	1	---	1	---	1	---	1	---
Body corpulence								
Thin (Z-score < −2)	1.24 [0.61–2.53]	0.54	1.27 [0.62–2.62]	0.51	0.61 [0.40–0.94]	0.024	0.66 [0.43–1.01]	0.059
Normal (Z-score ≥ −2 and <+1)	1	---	1	---	1	---	1	---
overweight (Z-score ≥ +1)	0.86 [0.55–1.35]	0.50	0.80 [0.50–1.29]	0.36	0.88 [0.72–1.09]	0.24	0.82 [0.66–1.03]	0.086
Type of school								
Private	1	---	1	---	1	---	1	---
Public	0.62 [0.33–1.16]	0.13	0.60 [0.31–1.17]	0.13	1.74 [1.34–2.21]	<0.0001	1.59 [1.16–2.18]	0.004
Father’s education								
Secondary or more schooling	1.13 [0.70–1.85]	0.60	1.12 [0.60–2.08]	0.72	1.00 [0.81–1.24]	0.97	1.10 [0.82–1.48]	0.53
Uncompleted secondary schooling	0.84 [0.51–1.38]	0.48	0.83 [0.48–1.42]	0.48	0.97 [0.80–1.19]	0.79	1.01 [0.79–1.29]	0.93
No formal schooling or Primary schooling	1	---	1	---	1	---	1	---
Mother’s education								
Secondary or more schooling	1.11 [0.73–1.69]	0.61	1.10 [0.64–1.89]	0.72	0.88 [0.70–1.11]	0.29	0.84 [0.92–1.14]	0.26
Uncompleted secondary schooling	1.18 [0.70–2.00]	0.53	1.26 [0.71–2.21]	0.43	0.96 [0.78–1.18]	0.72	0.96 [0.74–1.23]	0.73
No formal schooling or Primary schooling	1	---	1	---	1	--	1	---
Father’s occupation								
Middle/upper executive	1	---	1	---	1	---	1	--
Worker/employee	0.88 [0.58–1.32]	0.52	0.83 [0.48–1.42]	0.48	0.81 [0.64–1.03]	0.085	0.95 [0.74–1.22]	0.67
Not working	0.84 [0.42–1.67]	0.61	0.69 [0.34–1.40]	0.78	0.94 [0.57–1.52]	0.78	1.13 [0.67–1.91]	0.63
Mother’s occupation								
Middle/upper executive	1.04 [0.55–1.99]	0.89	1.04 [0.55–1.95]	0.41	1.29 [1.02–1.62]	0.036	1.18 [0.91–1.52]	0.076
Worker/employee	0.97 [0.49–1.90]	0.93	1.08 [0.57–2.05]	0.81	0.93 [0.71–1.2]	0.58	0.93 [0.70–1.24]	
Not working	1	---	1	---	1	---	1	---
Household economic level								
Low	1.41 [0.89–2.22]	0.14	1.57 [0.94–2.61]	0.083	0.81 [0.63–1.05]	0.11	0.96 [0.71–1.29]	0.79
Middle	0.89 [0.47–1.69]	0.73	0.91 [0.46–1.80]	0.79	0.84 [0.68–1.06]		0.96 [0.76–1.21]	0.72
High	1	---	1	---	1	---	1	---

^1^ Iron deficiency. ^2^ Odds Ratio. ^3^ 95% confidence interval. ^4^ Raw or adjusted *p*-value for logistic regression models accounting for the survey design across variable categories.

## Data Availability

The datasets presented in this article are not readily available because the data belongs to the Ministry of Health. Requests to access the datasets should be directed to jalila.elati@yahoo.fr.

## References

[1] Stevens G.A., Beal T., Mbuya M.N.N., Luo H., Neufeld L.M., Addo O.Y., Adu-Afarwuah S., Alayón S., Bhutta Z., Brown K.H. (2022). Micronutrient Deficiencies among Preschool-Aged Children and Women of Reproductive Age Worldwide: A Pooled Analysis of Individual-Level Data from Population-Representative Surveys. Lancet Glob. Health.

[2] Black R.E., Black R.E., Singhal A., Uauy R. (2014). Global Distribution and Disease Burden Related to Micronutrient Deficiencies. Nestlé Nutrition Institute Workshop Series.

[3] Chandra R. (2002). Nutrition and the Immune System from Birth to Old Age. Eur. J. Clin. Nutr..

[4] El Ati-Hellal M., Hellal F., Prieto M.A., Otero P. (2022). Food Supplementation with Vitamins and Minerals: An Overview. Natural Food Additives.

[5] Safiri S., Amiri F., Karamzad N., Sullman M.J.M., Kolahi A.-A., Abdollahi M. (2025). Burden and Trends of Dietary Iron Deficiency in the Middle East and North Africa Region, 1990–2021. Front. Nutr..

[6] Andriastuti M., Ilmana G., Nawangwulan S.A., Kosasih K.A. (2020). Prevalence of Anemia and Iron Profile among Children and Adolescent with Low Socio-Economic Status. Int. J. Pediatr. Adolesc. Med..

[7] Pasricha S.-R., Tye-Din J., Muckenthaler M.U., Swinkels D.W. (2021). Iron Deficiency. Lancet.

[8] NIN (1978). Enquête Nationale de Nutrition 1973–1975.

[9] INNTA (2000). Tunisian National Nutrition Survey 1996-1997.

[10] INNTA, UNICEF (2002). Anémies En Tunisie: Causes et Mesures D’Interventions.

[11] INSP, MS, OMS (2016). Tunisian Health Examination Survey.

[12] Halterman J.S., Kaczorowski J.M., Aligne C.A., Auinger P., Szilagyi P.G. (2001). Iron Deficiency and Cognitive Achievement Among School-Aged Children and Adolescents in the United States. Pediatrics.

[13] Bruner A.B., Joffe A., Duggan A.K., Casella J.F., Brandt J. (1996). Randomised Study of Cognitive Effects of Iron Supplementation in Non-Anaemic Iron-Deficient Adolescent Girls. Lancet.

[14] World Food Programme (WFP) (2024). State of School Feeding Worldwide 2024.

[15] World Health Organisation (1995). Physical Status: The Use of and Interpretation of Anthropometry, Report of a WHO Expert Committee.

[16] De Onis M., Dasgupta P., Saha S., Sengupta D., Blössner M. (2001). The National Center for Health Statistics Reference and the Growth of Indian Adolescent Boys. Am. J. Clin. Nutr..

[17] Onis M.d., World Health Organisation (2006). Length/Height-for-Age, Weight-for-Age, Weight-for-Length, Weight-for-Height and Body Mass Index-for-Age; Methods and Development.

[18] Morabia A., Bernstein M., Kumanyika S., Sorenson A., Mabiala I., Prodolliet B., Rolfo I., Luong B.L. (1994). Développement et validation d’un questionnaire alimentaire semi-quantitatif à partir d’une enquête de population. Soz. Präventivmedizin SPM.

[19] Dogui D., Doggui R., El Ati J., El Ati-Hellal M. (2021). Association between Overweight and Diet Diversity Score: A Cross-Sectional Study Conducted among Tunisian Children. Children.

[20] Dogui D., Doggui R., Al-Jawaldeh A., El Ati J., El Ati-Hellal M. (2022). Ultra-Processed Foods Are the Major Sources of Total Fat, Saturated and Trans-Fatty Acids among Tunisian Preschool and School Children: A Cross-Sectional Study. Children.

[21] Galobardes B. (2006). Indicators of Socioeconomic Position (Part 2). J. Epidemiol. Community Health.

[22] Traissac P., Martin-Prevel Y. (2012). Alternatives to Principal Components Analysis to Derive Asset-Based Indices to Measure Socio-Economic Position in Low- and Middle-Income Countries: The Case for Multiple Correspondence Analysis. Int. J. Epidemiol..

[23] Aounallah-Skhiri H., Traissac P., El Ati J., Eymard-Duvernay S., Landais E., Achour N., Delpeuch F., Romdhane H.B., Maire B. (2011). Nutrition Transition among Adolescents of a South-Mediterranean Country: Dietary Patterns, Association with Socio-Economic Factors, Overweight and Blood Pressure. A Cross-Sectional Study in Tunisia. Nutr. J..

[24] Hinnouho G.-M., Barffour M.A., Wessells K.R., Brown K.H., Kounnavong S., Chanhthavong B., Ratsavong K., Kewcharoenwong C., Hess S.Y. (2018). Comparison of Haemoglobin Assessments by HemoCue and Two Automated Haematology Analysers in Young Laotian Children. J. Clin. Pathol..

[25] World Health Organization (2011). Haemoglobin Concentrations for the Diagnosis of Anaemia and Assessment of Severity|Vitamin and Mineral Nutrition Information System.

[26] World Health Organization, World Health Organization (2024). Guideline on Haemoglobin Cutoffs to Define Anaemia in Individuals and Populations.

[27] Erhardt J.G., Estes J.E., Pfeiffer C.M., Biesalski H.K., Craft N.E. (2004). Combined Measurement of Ferritin, Soluble Transferrin Receptor, Retinol Binding Protein, and C-Reactive Protein by an Inexpensive, Sensitive, and Simple Sandwich Enzyme-Linked Immunosorbent Assay Technique. J. Nutr..

[28] Engle-Stone R., Nankap M., Ndjebayi A.O., Erhardt J.G., Brown K.H. (2013). Plasma Ferritin and Soluble Transferrin Receptor Concentrations and Body Iron Stores Identify Similar Risk Factors for Iron Deficiency but Result in Different Estimates of the National Prevalence of Iron Deficiency and Iron-Deficiency Anemia among Women and Children in Cameroon. J. Nutr..

[29] World Health Organization (2020). WHO Guideline on Use of Ferritin Concentrations to Assess Iron Status in Individuals and Populations.

[30] Thurnham D.I., McCabe L.D., Haldar S., Wieringa F.T., Northrop-Clewes C.A., McCabe G.P. (2010). Adjusting Plasma Ferritin Concentrations to Remove the Effects of Subclinical Inflammation in the Assessment of Iron Deficiency: A Meta-Analysis. Am. J. Clin. Nutr..

[31] Korn E.L., Graubard B.I. (1999). Analysis of Health Surveys.

[32] McLean E., Cogswell M., Egli I., Wojdyla D., De Benoist B. (2009). Worldwide Prevalence of Anaemia, WHO Vitamin and Mineral Nutrition Information System, 1993–2005. Public Health Nutr..

[33] Al-Jawaldeh A., Haq A., Ahmed K.M., Al-Jawaldeh H., Kari K.E., Nasreddine L., Afifi M., Hage M.O.E., Barham R., Khairy S. (2024). Anemia Among Children and Women in the Eastern Mediterranean Region: A Technical Guide. https://osf.io/yg6js/download/?format=pdf.

[34] Ati J.E., Lefèvre P., Béji C., Ben Rayana C., Gaigi S., Delpeuch F. (2008). Aetiological Factors and Perception of Anaemia in Tunisian Women of Reproductive Age. Public Health Nutr..

[35] Werner R., Luo H., Liu L., Wang Y., Geng J., Ko Y.-A., Suchdev P.S., Addo Y., Bhutta Z.A., Temple V. (2025). Micronutrients Associated with Anemia in School-Age Children and Adolescents 2005–2018: Biomarkers Reflecting Inflammation and Nutritional Determinants of Anemia (BRINDA) Project. Curr. Dev. Nutr..

[36] Iglesias Vázquez L., Valera E., Villalobos M., Tous M., Arija V. (2019). Prevalence of Anemia in Children from Latin America and the Caribbean and Effectiveness of Nutritional Interventions: Systematic Review and Meta–Analysis. Nutrients.

[37] Wu J., Hu Y., Li M., Chen J., Mao D., Li W., Wang R., Yang Y., Piao J., Yang L. (2019). Prevalence of Anemia in Chinese Children and Adolescents and Its Associated Factors. Int. J. Environ. Res. Public Health.

[38] Kundu S., Alam S.S., Mia M.A.-T., Hossan T., Hider P., Khalil M.I., Musa K.I., Islam M.A. (2023). Prevalence of Anemia among Children and Adolescents of Bangladesh: A Systematic Review and Meta-Analysis. Int. J. Environ. Res. Public Health.

[39] Tezera R., Sahile Z., Yilma D., Misganaw E., Mulu E. (2018). Prevalence of Anemia among School-Age Children in Ethiopia: A Systematic Review and Meta-Analysis. Syst. Rev..

[40] Gosdin L., Tripp K., Mahama A.B., Quarshie K., Amoaful E.F., Selenje L., Sharma D., Jefferds M.E., Sharma A.J., Whitehead R.D. (2020). Predictors of Anaemia among Adolescent Schoolchildren of Ghana. J. Nutr. Sci..

[41] Tawfik A.A., Hanna E.T., Abdel-Maksoud A.M. (2015). Anemia and Iron Deficiency Anemia in Egypt. IOSR J. Pharm..

[42] Banerjee M., Bhatti B.V.K., Roy D., Tomo S. (2022). A National Survey of the Prevalence of Anemia and Obesity in Indian School Children. J. Community Hosp. Intern. Med. Perspect..

[43] Codjia P., Leyna G., Msola H., Kagaruki G., Mchau G., Lukindo T., Masumo R., Killel E., Salmin A., Said F.A. (2025). Determinants of Anemia among School Children and Adolescents in Zanzibar. medRxiv.

[44] Wah S.T., Yi Y.S., Khin A.A., Plabplueng C., Nuchnoi P. (2017). Prevalence of Anemia and Hemoglobin Disorders Among School Children in Myanmar. Hemoglobin.

[45] El Hioui M., Touhami Ah A.O., Aboussaleh Y., Rusinek S., Dik K., Soualem A. (2008). Iron Deficiency and Anaemia in Rural School Children in a Coastal Area of Morocco. Pak. J. Nutr..

[46] Wrottesley S.V., Mates E., Brennan E., Bijalwan V., Menezes R., Ray S., Ali Z., Yarparvar A., Sharma D., Lelijveld N. (2023). Nutritional Status of School-Age Children and Adolescents in Low- and Middle-Income Countries across Seven Global Regions: A Synthesis of Scoping Reviews. Public Health Nutr..

[47] El Khoury R., Sleilaty G., Gannagé-Yared M.-H. (2020). Prevalence of Iron Deficiency in Lebanese Schoolchildren. Eur. J. Clin. Nutr..

[48] Nik Shanita S., Siti Hanisa A., Noor Afifah A.R., Lee S.T., Chong K.H., George P., Norazida A.B., Budin S.B., Khouw I., Norimah A.K. (2018). Prevalence of Anaemia and Iron Deficiency among Primary Schoolchildren in Malaysia. Int. J. Environ. Res. Public Health.

[49] Zheng H., Long W., Tan W., Yang C., Cao M., Zhu Y. (2020). Anaemia, Iron Deficiency, Iron-Deficiency Anaemia and Their Associations with Obesity among Schoolchildren in Guangzhou, China. Public Health Nutr..

[50] López-Ruzafa E., Vázquez-López M.A., Galera-Martínez R., Lendínez-Molinos F., Gómez-Bueno S., Martín-González M. (2021). Prevalence and Associated Factors of Iron Deficiency in Spanish Children Aged 1 to 11 Years. Eur. J. Pediatr..

[51] Visser M., Van Zyl T., Hanekom S.M., Baumgartner J., Van Der Hoeven M., Taljaard-Krugell C., Smuts C.M., Faber M. (2021). Associations of Dietary Diversity with Anaemia and Iron Status among 5- to 12-Year-Old Schoolchildren in South Africa. Public Health Nutr..

[52] World Health Organisation (2007). Conclusions and Recommendations of the WHO Consultation on Prevention and Control of Iron Deficiency in Infants and Young Children in Malaria-Endemic Areas. Food Nutr. Bull..

[53] Gardner W.M., Razo C., McHugh T.A., Hagins H., Vilchis-Tella V.M., Hennessy C., Taylor H.J., Perumal N., Fuller K., Cercy K.M. (2023). Prevalence, Years Lived with Disability, and Trends in Anaemia Burden by Severity and Cause, 1990–2021: Findings from the Global Burden of Disease Study 2021. Lancet Haematol..

[54] World Health Organization, World Health Organization (2004). Vitamin and Mineral Requirements in Human Nutrition.

[55] Ahmad Fuzi S.F., Koller D., Bruggraber S., Pereira D.I., Dainty J.R., Mushtaq S. (2017). A 1-h Time Interval between a Meal Containing Iron and Consumption of Tea Attenuates the Inhibitory Effects on Iron Absorption: A Controlled Trial in a Cohort of Healthy UK Women Using a Stable Iron Isotope. Am. J. Clin. Nutr..

